# Editorial for the special issue ‘cell‐to‐cell’ and ‘cell‐to‐bioreactor’ interactions

**DOI:** 10.1002/elsc.202200062

**Published:** 2023-01-04

**Authors:** Ralf Takors

**Affiliations:** ^1^ Institut für Bioverfahrenstechnik Universität Stuttgart Stuttgart Germany

Shifting the current fossil economy to a carbon neutral supply is an enormous challenge. Current geopolitical issues that severely endanger long‐term established fossil supply chains function as catalysts fostering novel solutions. In this context, the goals for establishing a resilient economy meet climate protection demands for reducing the human footprint in atmosphere. Biotechnological processes for producing fine chemicals and commodities by utilizing renewable resources may play an important role to make the transformation to a circular economy happen.

Bioprocesses already successfully documented their outstanding quality not only to compete with established fossil routes but also to complement and even to replace them in industrial practice. Long‐term established examples comprise the microbial production of amino acids, organic acids, technical enzymes, food additives, active pharma ingredients … and many more.

Interestingly enough, mono‐cultures are used, predominately. This reflects the steady improvement of molecular tools for efficiently manipulating microbes to produce the molecule of interests. However, the question may arise whether mono‐cultures should be the first choice for developing novel bioprocesses. Often enough product formation demands for precursors, reduction equivalents, energy demands, etc. that contradict cellular needs for growth and maintenance. Furthermore, the genetic engineering of hosts may well reach technical limits if targeted product formation opposes the lifestyle of the cells. Additionally, many examples for the production of antibiotics are known outlining that antibiotic production only starts in the presence of another interacting strain.

Consequently, the implementation of co‐cultures with well‐equilibrated interactions is a promising approach for establishing a new generation of bioproduction processes. Accordingly, this topic (cell‐to‐cell interactions) is highlighted in the current special issue and is also a central theme in the priority program ‘InterZell SPP2170’ of the German Science Foundation (DFG) that co‐fuels the special issue.

Once developed in the labs, novel bioprocesses should find their way into large‐scale bioreactors to translate innovation into practice. Often enough the so‐called scale‐up reveals the deterioration of key performance criteria such as titer, rates, and yield (TRY). This basically reflects cellular responses on mixing heterogeneities that inevitably occur in industrial scale. For mitigating related performance losses profound research is necessary. Consequently, the special issue also covers related studies (cell‐to‐bioreactor interactions) analyzing microbial responses in detail and developing novel scale‐down devices.

